# Traumatic Brain Injury, Seizures, and Cognitive Impairment Among Older Adults

**DOI:** 10.1001/jamanetworkopen.2024.26590

**Published:** 2024-08-08

**Authors:** Yiqi Zhu, Jonathan Williams, Kebede Beyene, Jean-Francois Trani, Ganesh M. Babulal

**Affiliations:** 1 School of Social Work, Adelphi University, Garden City, New York; 2 Department of Neurology, Washington University School of Medicine, St Louis, Missouri; 3 Department of Pharmaceutical and Administrative Sciences, University of Health Sciences and Pharmacy in St Louis, St Louis, Missouri; 4 National Conservatory of Arts and Crafts, Paris, France; 5 Institute of Public Health, Washington University School of Medicine, St Louis, Missouri; 6 Department of Psychology, Faculty of Humanities, University of Johannesburg, Johannesburg, South Africa; 7 Brown School of Social Work, Washington University in St Louis, St Louis, Missouri; 8 Department of Clinical Research and Leadership, The George Washington University School of Medicine and Health Sciences, Washington, DC

## Abstract

**Importance:**

Traumatic brain injury (TBI), seizures, and dementia increase with age. There is a gap in understanding the associations of TBI, seizures, and medications such as antiseizure and antipsychotics with the progression of cognitive impairment across racial and ethnic groups.

**Objective:**

To investigate the association of TBI and seizures with the risk of cognitive impairment among cognitively normal older adults and the role of medications in moderating the association.

**Design, Setting, and Participants:**

This multicenter cohort study was a secondary analysis of the Uniform Data Set collected between June 1, 2005, and June 30, 2020, from the National Alzheimer’s Coordination Center. Statistical analysis was performed from February 1 to April 3, 2024. Data were collected from participants from 36 Alzheimer’s Disease Research Centers in the US who were 65 years or older at baseline, cognitively normal at baseline (Clinical Dementia Rating of 0 and no impairment based on a presumptive etiologic diagnosis of AD), and had complete information on race and ethnicity, age, sex, educational level, and apolipoprotein E genotype.

**Exposure:**

Health history of TBI, seizures, or both conditions.

**Main Outcomes and Measures:**

Progression to cognitive impairment measured by a Clinical Dementia Rating greater than 0.

**Results:**

Among the cohort of 7180 older adults (median age, 74 years [range, 65-102 years]; 4729 women [65.9%]), 1036 were African American or Black (14.4%), 21 were American Indian or Alaska Native (0.3%), 143 were Asian (2.0%), 332 were Hispanic (4.6%), and 5648 were non-Hispanic White (78.7%); the median educational level was 16.0 years (range, 1.0-29.0 years). After adjustment for selection basis using propensity score weighting, seizure was associated with a 40% higher risk of cognitive impairment (hazard ratio [HR], 1.40; 95% CI, 1.19-1.65), TBI with a 25% higher risk of cognitive impairment (HR, 1.25; 95% CI, 1.17-1.34), and both seizure and TBI were associated with a 57% higher risk (HR, 1.57; 95% CI, 1.23-2.01). The interaction models indicated that Hispanic participants with TBI and seizures had a higher risk of cognitive impairment compared with other racial and ethnic groups. The use of antiseizure medications (HR, 1.23; 95% CI, 0.99-1.53), antidepressants (HR, 1.32; 95% CI, 1.17-1.50), and antipsychotics (HR, 2.15; 95% CI, 1.18-3.89) was associated with a higher risk of cognitive impairment, while anxiolytic, sedative, or hypnotic use (HR, 0.88; 95% CI, 0.83-0.94) was associated with a lower risk.

**Conclusions and Relevance:**

This study highlights the importance of addressing TBI and seizures as risk factors for cognitive impairment among older adults. Addressing the broader social determinants of health and bridging the health divide across various racial and ethnic groups are essential for the comprehensive management and prevention of dementia.

## Key Points

**Question:**

Are traumatic brain injury (TBI) and seizures associated with cognitive impairment across racial and ethnic groups?

**Findings:**

In a cohort study of 7180 adults aged 65 years or older, TBI was associated with a 25% higher cognitive impairment risk, while seizures were linked to a 40% higher cognitive impairment risk; Hispanic adults with seizures and TBI had a greater risk of cognitive impairment than adults in other racial and ethnic groups. Antiseizure and antipsychotic medications were associated with a higher cognitive impairment risk.

**Meaning:**

This study suggests that addressing common social determinants of health may reduce the racial and ethnic disparities and also the risks of these 3 interlinked conditions.

## Teaser

This cohort study investigates the association of traumatic brain injury and seizures with the risk of cognitive impairment among cognitively normal older adults from diverse racial and ethnic groups and the role of medications in moderating the association.

## Introduction

Traumatic brain injury (TBI), seizures, and dementia are interconnected conditions that increase with age. Racial and ethnic minority groups are disproportionately affected and face higher comorbidity risks due to structural racism in social and medical systems.^1,2,3,4^ In addition, the associations between these 3 diseases are complicated by the prescription of psychiatric, seizure, and other medications to manage neurologic disorders and other comorbidities.^1,2^

Alzheimer disease and related dementias (ADRD) affect 5% of older adults aged 65 to 74 years, but that rate increases to 33.3% for those aged 85 years or older.^3^ Traumatic brain injury results in 800 000 annual emergency department visits, with a nonfatal TBI hospitalization rate of 155.9 per 100 000 older adults in the US.^5^ Epilepsy, defined as recurrent unprovoked or spontaneous seizures, is the third most common neurologic disease among older adults in the US after dementia and stroke.^6^ Approximately 25% to 30% of seizures are provoked by brain injuries or other conditions.^7^ Limited studies show that older adults with seizures and TBI often experience significant cognitive impairment.^8,9,10^

The number of individuals in the US with ADRD is projected to reach 14 million by 2050. African American and Hispanic individuals are 2 and 1.5 times more likely to have ADRD, respectively, compared with non-Hispanic White (hereafter, *White*) individuals.^3^ Similarly, TBI is a significant cause of death and disability in the US, with some racial and ethnic minority groups experiencing higher mortality^11^ and worse functional outcomes after TBI.^12^ American Indian and Alaska Native individuals have the highest rates of TBI-related deaths and hospitalization but significantly lower rates of rehabilitation use, while African American individuals face higher TBI risks from violence due to structural racism.^11,12,13,14,15,16^ Racial and ethnic minority groups were also disproportionally affected by seizures, with African American individuals having the highest seizure frequencies,^17,18^ hospitalizations, and emergency department visits,^14^ along with lower adherence to antiseizure medications and lower epilepsy surgery rates compared with White individuals. African American individuals with epilepsy also had a higher mortality rate compared with individuals from other racial and ethnic groups.^19,20^

The associations between seizures, TBI, and dementia are understudied, especially among diverse racial and ethnic groups. Emerging evidence suggests potential comorbidity and mutual associations among these diseases, but, to our knowledge, few studies have examined racial and ethnic differences in disease progression. A 2017 systematic review of 32 observational studies found that any type of brain injury significantly increased ADRD risk.^21^ However, only 3 studies reported TBI prevalence and its associations with dementia among different races and ethnicities in the US.^10,22,23^ A recent systematic review^24^ identified only 3 studies^25,26,27^ linking seizure or epilepsy with an increased risk of dementia. Two studies^25,27^ used community cohorts from limited regions, while the other focused on a primarily male veteran population—both indicating potential selection bias.^26^ A recent study found that ADRD risk was higher among participants with posttraumatic epilepsy than those with only head injury or seizure.^28^

Findings on the association of antiseizure medications with cognitive impairment and ADRD are mixed.^29^ A meta-analysis identified 9 studies,^30^ 6 of which found an increased ADRD risk associated with exposure to antiseizure medications,^31,32,33,34,35,36^ while the remaining studies found nonsignificant associations.^32,37,38,39^ The progression of cognitive impairment can be associated with the medications used to treat seizures, psychiatric symptoms, pain, and other neurologic conditions. However, the association of these medications with ADRD risk is understudied and requires further examination across diverse racial and ethnic groups.

This study used data in the Uniform Data Set (UDS) from the National Alzheimer’s Coordination Center (NACC) to investigate the association of TBI and seizure with cognitive impairment risk among cognitively normal older adults from diverse racial and ethnic groups. The study also examined the role of medications in the progression from cognitive normality to impairment among older adults with TBI and seizures. The hypotheses stipulated that (1) the presence of TBI and/or seizures would be associated with higher ADRD risk across all participants but would be worse for participants from racial and ethnic minority groups and (2) antiseizure and psychiatric medication use would moderate the association of TBI and/or seizure with ADRD risk.

## Methods

### Study Design and Settings

This cohort study was a secondary data analysis. We examined UDS data (data freeze: June 1, 2005, to June 30, 2020) collected from 36 Alzheimer’s Disease Research Centers (ADRC) and curated by the NACC in Seattle, Washington. All participants provided written informed consent, and institutional review board approval was obtained at each ADRC.^40^ This data use was approved by the NACC and, because the data were deidentified, the study was exempted from institutional review board review by the Washington University in St Louis Human Resources Protection Office. This report followed the Strengthening the Reporting of Observational Studies in Epidemiology (STROBE) reporting guideline for cohort studies.

### Participants

Based on the UDS data, participants were included if they were (1) aged 65 years or older at the first visit, (2) cognitively normal at baseline as indicated by a Clinical Dementia Rating (CDR)^41^ of 0 and lack of impairment based on a presumptive etiologic diagnosis of Alzheimer disease (“NACCALZD” variable in the NACC dataset = 8, indicating the absence of a presumptive etiologic diagnosis of Alzheimer disease; no probable and possible Alzheimer disease based on NINCDS-ADRDA [National Institute of Neurological and Communicative Diseases and Stroke/Alzheimer's Disease and Related Disorders Association] criteria^42^ and NIA-AA [National Institute on Aging–Alzheimer's Association] criteria^42^), and (3) had complete information on race and ethnicity, age, sex, educational level, and apolipoprotein E (APOE) genotype. A cohort of 7180 participants was examined until attrition (dropout or death) or the first instance of cognitive impairment progression (CDR >0).

### Variables and Measurement

The outcome variable was cognitive impairment progression. Traumatic brain injury and seizures as variables of interest were determined by the clinician’s diagnostic judgment after an annual interview about medical history and clinical evaluation with participants. The UDS provides information on the frequency and severity of TBI, not seizures. The frequency of TBI was recorded as single and multiple or repeated, and the severity of TBI was classified as a brief loss of consciousness, extended loss of consciousness, chronic loss of consciousness, or without loss of consciousness.

Data on race and ethnicity were collected at the first visit and were based on self-reported ethnicity (Hispanic or non-Hispanic) and racial categories (African American or Black, American Indian or Alaska Native, Asian, Native Hawaiian or Other Pacific Islander, White, other [a choice made available by NACC to individual participants for their self-identified race or ethnicity; <10 participants chose this choice and filled out the data; based on their answers, we organized them into 1 of the 5 racial categories], or unknown). The groups used in this study were African American or Black, American Indian or Alaska Native, Asian, Hispanic, and White. No participants identified as Native Hawaiian or Other Pacific Islander. Years of education, age, sex, and APOE status were used as covariates.

Medication categories were derived by the NACC using Multum-Lexicomp therapeutic drug categories. We examined the following drug categories provided by the NACC UDS: antidepressants, anxiolytics, sedatives or hypnotic agents, antipsychotics, and antiseizure medications. We also created an antiseizure medication category based on the lists provided by the American Epilepsy Society while excluding the medications that overlapped with anxiolytics (ie, clonazepam, diazepam, lorazepam, and midazolam) (eTable 1 in Supplement 1).^43^ These medication classes were chosen for their relevance in managing symptoms or sequelae associated with cognitive impairment, seizures, and TBI. Data on the dose, route, frequency, and duration of the drug groups were unavailable.^40^

### Statistical Analysis

Statistical analysis was conducted from February 1 to April 3, 2024. We conducted survival analyses to examine the associations of TBI, seizure, or both with the progression of cognitive impairment. Given the imbalanced characteristics among participants, such as age and educational level, it was essential to adjust for sample selection bias to ensure the independence of error terms from observed covariates. We used propensity score matching to address this imbalance, using the inverse probability treatment weighting (IPTW) technique. In addition, the interaction terms with the racial and ethnic group and the presence of TBI and/or seizures were used in both IPTW-unadjusted and IPTW-adjusted models to examine the differential associations of TBI and/or seizures with cognitive impairment.

To create the IPTW, we used the Olmos and Govindasamy procedure.^44^ Logistic regression was used to estimate the propensity scores based on age, sex, race and ethnicity, and educational level. These scores represent the probability of cognitive impairment based on the demographic factors. The inverse of these propensity scores was then used as weights in the survival analysis to reduce the selection bias.

To assess the potential associations of medication with cognitive impairment progression, each medication category was incorporated into a survival model alongside seizures and/or TBI and demographic control variables. Moderating effects were evaluated using interaction terms between medication categories and TBI and/or seizures. Due to the small number of patients taking antipsychotics and antiseizure medications, we did not analyze drugs at the subclass level. However, for antidepressants, a sensitivity analysis was conducted by distinguishing between first-generation and second-generation antidepressant classes (eTable 2 in Supplement 1), although most users were prescribed second-generation antidepressants (eTable 9 in Supplement 1).^45^

To evaluate the robustness of the association of TBI and/or seizures with the risk of cognitive impairment progression, we conducted sensitivity analyses using 2 distinct methods: optimal full matching and the Fine-Gray model.^46^ Optimal full matching, a specific form of propensity score matching, matched variable numbers of treated and control participants to minimize the overall distance between propensity scores within matched sets.^47,48,49,50^ The Fine-Gray model was used to adjust for death as a competing risk factor.^46^ To account for potential risk factors, we tested the models with severity and frequency of TBI, the number of neuropsychiatric symptoms based on the Neuropsychiatric Inventory–Questionnaire, and the ADRC center as approximate geographic location as sensitivity analysis. All *P* values were from 2-sided tests and results were deemed statistically significant at *P* < .05.

## Results

In this cohort study of 7180 participants (median age, 74 years [range, 65-102 years]; 4729 women [65.9%] and 2451 men [34.1%]), 1036 were African American or Black (14.4%), 21 were American Indian or Alaska Native (0.3%), 143 were Asian (2.0%), 332 were Hispanic (4.6%), and 5648 were non-Hispanic White (78.7%); the median educational level was 16.0 years (range, 1.0-29.0 years) (Table 1). Median follow-up for the total sample was 4.0 years (range, 0.1-14.7 years); at the end of the study, 2020 participants (28.1%) progressed to cognitive impairment with a median follow-up of 3.2 years (range, 0.2-14.2 years). Follow-up was significantly shorter for those who progressed to a CDR greater than 0 (median, 3.2 years; range, 0.2-14.2 years) than for those who did not (median, 4.2 years; range, 0.5-14.7 years). In the total sample, 907 participants (12.6%) reported a TBI, 104 (1.5%) reported seizure, and 42 (0.6%) reported both. The rate of diagnosis of seizure and TBI was unevenly distributed among different racial and ethnic groups. For example, of 5648 White participants, 774 (13.7%) had a diagnosis of TBI, and 82 (1.5%) had a diagnosis of seizure; of 1036 African American or Black participants, 74 (7.1%) had a diagnosis of TBI, and 17 (1.6%) had a diagnosis of seizure. There were only 21 American Indian or Alaska Native participants, but 3 (14.3%) had a diagnosis of TBI.

**Table 1.** Baseline Characteristics of Participants

Characteristic	CDR = 0 (n = 5160)	CDR >0 (n = 2020)	*P* value^a^	African American or Black (n = 1036)	American Indian or Alaska Native (n = 21)	Asian (n = 143)	Hispanic (n = 332)	White (n = 5648)
CDR >0, No. (%)	0	2020 (100)	NA	229 (22.1)	8 (38.1)	39 (27.3)	99 (29.8)	1645 (29.1)
Diagnosis, No. (%)								
Neither	4477 (86.8)	1650 (81.7)	<.001	940 (90.7)	18 (85.7)	127 (88.8)	283 (85.2)	4759 (84.3)
Seizure alone	60 (1.2)	44 (2.2)		17 (1.6)	0	2 (1.4)	3 (0.9)	82 (1.5)
TBI alone	595 (11.5)	312 (15.4)		74 (7.1)	3 (14.3)	14 (9.8)	42 (12.7)	774 (13.7)
Seizure and TBI	28 (0.5)	14 (0.7)		5 (0.5)	0	0	4 (1.2)	33 (0.6)
Sex, No. (%)								
Male	1706 (33.1)	745 (36.9)	.002	207 (20.0)	4 (19.0)	52 (36.4)	97 (29.2)	2091 (37.0)
Female	3454 (66.9)	1275 (63.1)		829 (80.0)	17 (81.0)	91 (63.6)	235 (70.8)	3557 (63.0)
Age, median (range), y	72.0 (65.0-101.0)	77.0 (65.0-102.0)	<.001	72.0 (65.0-101.0)	70.0 (66.0-83.0)	73.0 (65.0-91.0)	72.0 (65.0-90.0)	74.0 65.0-102.0)
Educational level, median (range), y	16.0 (1.0-29.0)	16.0 (2.0-29.0)	<.001	14.0 (2.0-24.0)	16.0 (12.0-18.0)	18.0 (8.0-22.0)	14.0 (2.0-24.0)	16.0 (1.0-29.0)
APOE status, No. (%)								
*APOE ε*4 negative	3742 (72.5)	1369 (67.8)	<.001	674 (65.1)	15 (71.4)	126 (88.1)	247 (74.4)	4049 (71.7)
*APOE ε*4 positive	1418 (27.5)	651 (32.2)		362 (34.9)	6 (28.6)	17 (11.9)	85 (25.6)	1599 (28.3)
GDS score, mean (SD) (n = 7026)	0 (0-15.0)	1.0 (0-15.0)	<.001	0 (0-11.0)	1.0 (0-5.0)	1.0 (0-10.0)	1.0 (0-13.0)	0 (0-15.0)
NPIQ score, median (range) (n = 6662)	0 (0-17.0)	0 (0-19.0)	<.001	0 (0-13.0)	0 (0-6.0)	0 (0-8.0)	0 (0-11.0)	0 (0-19.0)
MMSE score, median (range) (n = 5666)	29.0 (17.0-30.0)	29.0 (18.0-30.0)	<.001	29.0 (17.0-30.0)	27.0 (24.0-30.0)	29.0 (23.0-30.0)	29.0 (21.0-30.0)	29.0 (21.0-30.0)
Follow-up, median (range), y	4.2 (0.5-14.7)	3.2 (0.2-14.2)	<.001	4.1 (0.6-13.8)	2.3 (1.1-8.4)	3.2 (0.83-14.2)	3.1 (0.7-13.5)	4.0 (0.2-14.7)
Vital status, No. (%)								
Alive	4519 (87.6)	1385 (68.6)	<.001	900 (86.9)	20 (95.2)	138 (96.5)	306 (92.2)	4540 (80.4)
Dead	641 (12.4)	635 (31.4)		136 (13.1)	1 (4.8)	5 (3.5)	26 (7.8)	1108 (19.6)

Abbreviations: APOE, apolipoprotein E; CDR, Clinical Dementia Rating; GDS, Geriatrics Depression Scale; MMSE, Mini-Mental State Examination; NA, not applicable; NPIQ, Neuropsychiatric Inventory Questionnaire; TBI, traumatic brain injury.

^a^ *P* values were generated by χ^2^ tests for categorical variables, and Kolmogorov-Smirnov tests for continuous variables.

Participants who progressed to CDR greater than 0 also had significantly higher scores on the Geriatric Depression Scale than those who did not (median, 1 [0-15] vs 0 [0-15]; *P* < .001). In addition, participants who progressed to cognitive impairment were significantly older, male, and had fewer years of education (Table 1). Notable differences in demographic characteristics were observed by race and ethnicity of participants. White participants were older and exhibited higher rates of TBI and seizure, African American participants were more likely to be female, and Asian participants had the highest level of education.

Kaplan-Meier analysis indicated that seizure and TBI were associated with higher risks of cognitive impairment (Figure). Before propensity score matching, the unadjusted model showed that seizure was associated with a 68% higher risk of progression to a CDR greater than 0 (hazard ratio [HR], 1.68; 95% CI, 1.24-2.26), while TBI was associated with a 29% higher risk (HR, 1.29; 95% CI, 1.14-1.46) (Table 2). African American participants had a 16% lower risk of progression to a CDR greater than 0 (HR, 0.84; 95% CI, 0.73-0.97), while Hispanic participants had a 46% higher risk of a CDR greater than 0 compared with White participants (HR, 1.46; 95% CI, 1.19-1.80). The difference between Asian and White participants was not significant.

**Figure.**
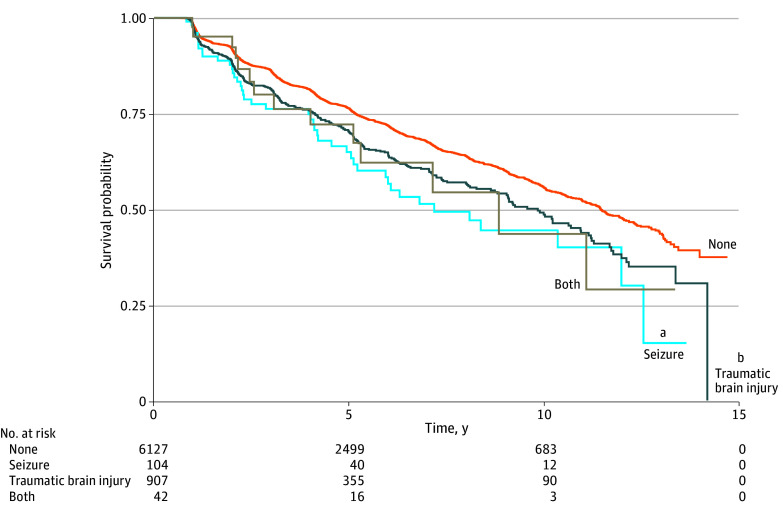
Kaplan-Meier Curve of Time to Cognitive Impairment Across Traumatic Brain Injury and Seizure Groups ^a^*P* < .001. ^b^*P* = .002.

**Table 2.** Associations Between Traumatic Brain Injury, Seizure, and Progression to Incident Cognitive Impairment

Variable	Hazard ratio (95% CI)	
	Model without PSW^a^	Model with PSW^a^
Condition		
Traumatic brain injury	1.29 (1.14-1.46)^b^	1.25 (1.17-1.34)^b^
Seizure	1.68 (1.24-2.26)^b^	1.40 (1.19-1.65)^c^
Both	1.41 (0.83-2.40)	1.57 (1.23-2.01)^d^
Race and ethnicity		
African American or Black	0.84 (0.73-0.97)^d^	1.10 (1.03-1.18)
Asian	1.28 (0.93-1.76)	1.24 (1.05-1.46)
Hispanic	1.46 (1.19-1.80)^b^	1.24 (1.11-1.39)^d^
Non-Hispanic White	1 [Reference]	1 [Reference]
Sex		
Female	0.85 (0.77-0.93)^b^	0.91 (0.87-0.96)^d^
Male	1 [Reference]	1 [Reference]
Age	1.07 (1.07-1.08)^b^	1.02 (1.01-1.02)^b^
Educational level	0.98 (0.96-0.99)^c^	0.99 (0.98-1.00)
APOE ε4 posititve	1.52 (1.38-1.67)^b^	1.40 (1.33-1.47)^b^
Observations, No.	7159	7159
*R*^2^ Nagelkerke	0.078	0.045

Abbreviations: APOE, apolipoprotein E; PSW, propensity score weighting.

^a^ The inverse of propensity scores, which is estimated based on age, sex, race and ethnicity, and educational level. These scores represent the probability of each participant progressing to cognitive impairment based on their demographic characteristics. The inverse of these propensity scores was then used as weights (inverse probability treatment weighting) in the survival analysis to examine the associations of traumatic brain injury and/or seizure with progression of cognitive impairment with the balanced weights of demographic variables.

^b^ *P* < .001.

^c^ *P* < .01.

^d^ *P* < .05.

In the IPTW-adjusted model, seizure was associated with a 40% higher risk for progression (HR, 1.40; 95% CI, 1.19-1.65), TBI was associated with a 25% higher risk for progression (HR, 1.25; 95% CI, 1.17-1.34), and diagnoses of both TBI and seizures were associated with a 57% higher risk for progression (HR, 1.57; 95% CI, 1.23-2.01) (Table 2). Hispanic individuals were 24% more likely than White individuals to progress to a CDR greater than 0 (HR, 1.24; 95% CI, 1.11-1.39). Due to a small number of Asian participants, only 3 racial and ethnic groups were included in testing interaction effects. Significant associations were found among Hispanic participants with seizure in the unadjusted model and with TBI in the adjusted model (eTable 3 in Supplement 1).

In the unadjusted models, the addition of medications revealed that baseline antidepressant use was associated with a 32% increased risk of progressing to a CDR greater than 0 (HR, 1.32; 95% CI, 1.17-1.50) and antipsychotic use with a 115% increased risk (HR, 2.15; 95% CI, 1.18-3.89) (Table 3). Conversely, the use of anxiolytics, sedatives, or hypnotics was associated with a 12% reduced risk of cognitive impairment (HR, 0.88; 95% CI, 0.83-0.94). In IPTW-adjusted models, antiseizure medication was associated with a 33% increased risk of cognitive impairment (HR, 1.33; 95% CI, 1.19-1.49), and the direction and significance of the associations between medication use and the risk of progressing to a CDR greater than 0 remained consistent across other medication classes. Interaction terms between TBI and/or seizure and medications did not show significant associations when added to the models.

**Table 3.** Role of Medication on the Risk of Progression to Incident Cognitive Impairment

Medication	Hazard ratio (95% CI)						
	Progression to CDR >0	Seizure^a^	TBI	Seizure and TBI	African American or Black	Asian	Hispanic
**Models without PSW, medications**							
Antidepressant	1.32 (1.17-1.50)^b^	1.69 (1.25-2.29)^b^	1.28 (1.13-1.45)^b^	1.22 (0.69-1.50)	0.87 (0.71-1.01)	1.32 (0.95-1.82)	1.48 (1.20-1.82)^b^
Antipsychotic	2.15 (1.18-3.89)^c^	1.67 (1.23-2.26)^b^	1.28 (1.13-1.45)^b^	1.26 (0.71-2.22)	0.84 (0.73-0.98)^d^	1.27 (0.92-1.76)	1.46 (1.19-1.81)^b^
Anxiolytic, sedative, or hypnotic	0.88 (0.83-0.94)^b^	1.68 (1.23-2.27)^b^	1.29 (1.14-1.46)^b^	1.41 (0.83-2.38)	0.84 (0.72-0.96)^d^	1.28 (0.93-1.77)	1.46 (1.19-1.80)^b^
Antiseizure	1.23 (0.99-1.53)	1.64 (1.21-2.21)^c^	1.29 (1.15-1.46)^b^	1.34 (0.79-2.29)	0.85 (0.73-0.98)^d^	1.29 (0.93-1.77)	1.46 (1.19-1.80)^b^
**Model with PSW, medications**							
Antidepressant	1.29 (1.29-1.43)^b^	1.42 (1.10-1.82)^c^	1.25 (1.12-1.40)^b^	1.37 (0.69-2.15)	1.14 (1.01-1.29)^c^	1.27 (0.96-1.69)	1.26 (1.06-1.50)^c^
Antipsychotic	1.79 (1.12-2.85)^c^	1.41 (1.09-1.82)^c^	1.25 (1.12-1.39)^b^	1.44 (0.93-2.24)	1.11 (0.98-1.25)^c^	1.23 (0.93-1.63)	1.25 (1.05-1.48)^d^
Anxiolytic, sedative, or hypnotic	0.94 (0.89-0.99)^d^	1.39 (1.09-1.79)^c^	1.25 (1.12-1.39)^b^	1.57 (1.03-2.37)^d^	1.09 (0.97-1.24)	1.24 (0.94-1.64)	1.24 (1.05-1.47)^d^
Antiseizure	1.33 (1.19-1.49)^c^	1.35 (1.14-1.59)^d^	1.25 (1.18-1.34)^b^	1.48 (1.16-1.90)	1.10 (1.03-1.18)	1.24 (1.05-1.47)	1.24 (1.11-1.39)^d^

Abbreviations: CDR, Clinical Dementia Rating; PSW, propensity score weighting; TBI, traumatic brain injury.

^a^ Reference: neither seizure nor TBI.

^b^ *P* < .001.

^c^ *P* < .01.

^d^ *P* < .05.

Sensitivity analyses showed that neither TBI severity nor frequency was significantly associated with an increased risk of progressing to a CDR greater than 0 (eTable 4 in Supplement 1). However, in IPTW-adjusted sensitivity analyses, the use of both first-generation and second-generation antidepressant classes was associated with an increased risk of progressing to a CDR greater than 0 compared with nonuse (first-generation: HR, 1.21; 95% CI, 1.08-1.36; second-generation: HR, 1.30; 95% CI, 1.21-1.40) (eTable 5 in Supplement 1).

Adding centers and the numbers of reported neuropsychiatric symptoms did not change the results (eTables 6 and 7 in Supplement 1). The results using the Fine-Gray model were similar to the model without IPTW adjustment, and the full optimal matching model yielded similar results as those of IPTW-adjusted models (eTable 8 in Supplement 1).

## Discussion

Traumatic brain injury, seizure, and both conditions combined were associated with increased risk of cognitive impairment over a median follow-up of 4 years among a community cohort of healthy older adults. Racial and ethnic disparities were found when the risk of cognitive impairment was higher for Hispanic participants in the unadjusted model but lower for African American participants compared with White participants. The moderating effects of medication use on the associations of TBI and/or seizure with ADRD were not significant. However, medication use alone was significantly associated with progression to cognitive impairment. The *APOE* ε4 allele is a well-established risk factor for cognitive impairment progression and Alzheimer disease^51^; however, after TBI, it is also implicated in slower recovery,^52^ greater cognitive impairment in memory and attention,^53^ and greater inflammatory response.^54^ The results adjusting for *APOE* ε4 still showed TBI and seizures as being associated with a risk of progression to cognitive impairment.

The NACC dataset is one of the largest, well-characterized, longitudinal ADRD databases, with over 37 ADRCs throughout the US in the past 20 years.^40^ Evidence from this study underscores the importance of addressing TBI and seizures as risk factors for progression of cognitive impairment among older adults.

The disparity in progression of cognitive impairment, especially among Hispanic individuals with TBI, highlights the broader issue of health care inequalities.^55^ Studies found that while there was not a significant difference in severe TBI treatment in the emergency department, delayed diagnosis can occur with mild TBI.^14,56,57^ Health care utilization often intersects with discrimination and structural barriers for different groups.^58^ Hispanic patients often wait longer in the emergency department to see a physician and are more likely to leave without being seen compared with White patients.^56,57,59^

The disparity between other racial and ethnic groups was not significant after adjusting for selection biases, likely due to underdiagnosis of TBI, seizure, and cognitive impairment, as well as representation (eg, older, healthy, and well educated) of racial and ethnic groups across ADRCs.^60^ For example, 36.1% of White patients received a diagnosis of ADRD at their baseline visits across all ADRCs from 2005 to 2020 compared with 26.8% of African American patients.^61^ Population studies based on the Health and Retirement Study and Medicare showed that African American individuals are twice as likely to develop Alzheimer disease compared with White individuals.^62,63,64^ The different cognitive impairment incidences from the NACC also resulted from differential recruitment strategies across racial and ethnic groups.^65^

Traumatic brain injury and seizures were also underdiagnosed among medically underserved communities. Compared with White veterans, African American veterans had a 27% lower odds of TBI diagnosis among service members returning from Afghanistan and Iraq.^66^ Studies among the general population found no significant difference in treatment of severe TBI in the emergency department, but delayed diagnosis can occur with mild TBI.^14,56,57^

Our findings on the association between medication use and progression of cognitive impairment should be interpreted cautiously due to a lack of detailed information on dose, route, and frequency of intake. Most of the medication users in this study were White (eTable 9 in Supplement 1). Despite this limitation, significant associations of the use of antidepressants, antipsychotics, and antiseizure medications with risks of cognitive impairment were observed. First-generation antidepressants (eg, tricyclic antidepressants) and older antipsychotics (eg, haloperidol and chlorpromazine) have strong anticholinergic properties.^39,67^ These properties can block the action of acetylcholine, a neurotransmitter vital for learning, memory, and muscle activation, potentially leading to cognitive impairment.^67^ The consistency observed in sensitivity analyses, particularly with first-generation and second-generation antidepressants, underscores the potential role of cognitive decline. Antidepressants and antipsychotics are often prescribed for conditions such as depression and schizophrenia, which themselves are risk factors for cognitive impairment.^68^ Thus, the observed association may partially reflect the underlying conditions rather than the medication effects alone. Similarly, antiseizure medication can impair cognitive functions by reducing neuronal excitability or increasing inhibitory neurotransmission.^29^ These drugs often affect attention, vigilance, and psychomotor speed and may also influence other cognitive abilities.^69,70^

Conversely, the protective effect associated with anxiolytics, sedatives, and hypnotics could be due to their potential to reduce anxiety and improve sleep patterns, which are crucial for maintaining cognitive health. Improved sleep has been shown to mitigate amyloid deposition, a pathologic hallmark of Alzheimer disease.^71^ Overall, our findings highlight the complexities of managing TBI and seizures with associated neuropsychiatric conditions, underscoring the importance of careful medication selection.

### Limitations

Despite the large sample and longer follow-up period, this study has limitations. Data collection practices on cognitive impairment, TBI, and seizure vary across ADRCs, even though these data are analyzed as uniform variables. The medication data are inadequately characterized regarding dose, duration, adherence, and frequency. In addition, participants were recruited from diverse communities using different recruiting strategies, which may not represent the general population of older adults in the US.^72^

## Conclusions

In this cohort study involving community-dwelling older adults, significant associations of TBI and seizure with cognitive impairment were found, and higher risks were associated with medically underserved racial and ethnic minority groups. These results underscore the importance of addressing social determinants of health and reducing health disparities to manage and prevent cognitive impairment effectively. In addition, the use of anxiolytics, sedatives, or hypnotic agents was linked with a reduced risk of progression of cognitive impairment. However, further research is necessary to assess the association of various medications with the progression of cognitive impairment.

## Notes

Supplement 1. **eTable 1.** A List of Anti-Seizure Medications Used **eTable 2.** A List of Antidepressants and Their Categorizations **eTable 3.** The Interaction Effects of Seizure and TBI and Racial and Ethnic Groups and Progression to Incident Cognitive Impairment Using Unweighted Models **eTable 4.** Subanalysis Examining the Association Between Severity and Frequency of TBI and Cognitive Impairment Among Participants With TBI **eTable 5.** Associations Between Antidepressants Generation and Cognitive Impairment **eTable 6.** Examining Neuropsychiatric Symptoms for Sensitivity Analysis **eTable 7.** Examining NACC Center for Sensitivity Analysis **eTable 8.** Associations Between Seizure and TBI and Progression to Cognitive Impairment **eTable 9.** The Number of Users of Medications by Racial and Ethnic Groups

Supplement 2.
Data Sharing Statement
