# Supplementary material for: Traumatic Brain Injury, Seizures, and Cognitive Impairment Among Older Adults
Source: JAMA Netw Open. 2024 Aug 8;7(8):e2426590. doi: 10.1001/jamanetworkopen.2024.26590 (PMC11310819; doi:10.1001/jamanetworkopen.2024.26590)
Supplement: Supplement 1. — eTable 1. A List of Anti-Seizure Medications Used eTable 2. A List of Antidepressants and Their Categorizations eTable 3. The Interaction Effects of Seizure and TBI and Racial and Ethnic Groups and Progression to Incident Cognitive Impairment Using Unweighted Models eTable 4. Subanalysis Examining the Association Between Severity and Frequency of TBI and Cognitive Impairment Among Participants With TBI eTable 5. Associations Between Antidepressants Generation and Cognitive Impairment eTable 6. Examining Neuropsychiatric Symptoms for Sensitivity Analysis eTable 7. Examining NACC Center for Sensitivity Analysis eTable 8. Associations Between Seizure and TBI and Progression to Cognitive Impairment eTable 9. The Number of Users of Medications by Racial and Ethnic Groups [file jamanetwopen-e2426590-s001.pdf]

Supplementary Online Content

Zhu Y, Willaims J, Beyene K, Trani JF, Babulal GM. Traumatic brain injury, seizures, and cognitive impairment among older adults. *JAMA Netw Open.* 2024;7(8):e2426590. doi:10.1001/jamanetworkopen.2024.26590

eTable 1. A List of Anti-Seizure Medications Used
eTable 2. A List of Antidepressants and Their Categorizations
eTable 3. The Interaction Effects of Seizure and TBI and Racial and Ethnic Groups and Progression to Incident Cognitive Impairment Using Unweighted Models
eTable 4. Subanalysis Examining the Association Between Severity and Frequency of TBI and Cognitive Impairment Among Participants With TBI
eTable 5. Associations Between Antidepressants Generation and Cognitive Impairment
eTable 6. Examining Neuropsychiatric Symptoms for Sensitivity Analysis
eTable 7. Examining NACC Center for Sensitivity Analysis
eTable 8. Associations Between Seizure and TBI and Progression to Cognitive Impairment
eTable 9. The Number of Users of Medications by Racial and Ethnic Groups

This supplementary material has been provided by the authors to give readers additional information about their work.

© 2024 Zhu Y et al. *JAMA Network Open.*

eTable 1. A list of anti-seizure medications used

Drug code	**Drug name**
d00058	Carbamazepine
d00058	Carbamazepine-XR
d04557	Clobazam
d03833	Divalproex Sodium
d03833	Divalproex Sodium-ER
d00227	Ethosuximide
d03170	Felbamate
d00812	Fenfluramine
d03182	Gabapentin
d07349	Lacosamide
d03809	Lamotrigine
d04499	Levetiracetam
d04499	Levetiracetam XR
d04513	Oxcarbazepine
d00340	Phenobarbital
d00143	Phenytoin
d05508	Pregabalin
d00352	Primidone
d07069	Rufinamide
d04221	Tiagabine Hydrochloride
d04115	Topiramate
d04115	Topiramate XR
d00083	Valproic Acid
d04669	Vigabatrin
d04527	Zonisamide

© 2024 Zhu Y et al. *JAMA Network Open.*

eTable 2. A list of antidepressants and their categorizations

Category	Drug Class	Generic names
First-generation	Tricyclic antidepressants (TCAs)	Amitriptyline, Clomipramine, Desipramine, Doxepin, Imipramine, Nortriptyline, Amoxapine, Protriptyline, Trimipramine
First-generation	Monoamine oxidase inhibitors (MAOIs)	Tranylcypromine, Phenelzine, Selegiline, Isocarboxazid
First-generation	Other	Maprotiline, Trazodone
Second-generation	Selective Serotonin Re-uptake inhibitors(SSRIs)	Fluoxetine, Fluvoxamine, Paroxetine, Sertraline, Citalopram, Escitalopram, Vilazodone,
Second-generation	Serotonin and Norepinephrine Reuptake Inhibitors (SNRIs)	Venlafaxine, Duloxetine, Levomilnacipran, desvenlafaxine
Second-generation	5-HT2 receptor antagonists	Nefazodone
Second-generation	Dopamine re-uptake inhibitors	Bupropion
Second-generation	Other	Mirtazapine

© 2024 Zhu Y et al. *JAMA Network Open.*

eTable 3. The interaction effects of seizure and TBI and racial and ethnic groups and progression to incident cognitive impairment using unweighted models

	Model without PSW	Model with PSW
Predictors	*HR (CI)*	*HR(CI)*
Ref: Neither seizure nor TBI		
Seizure	1.59 ** (1.14 – 2.22)	1.34 * (1.11 – 1.62)
TBI	1.24 ** (1.09 – 1.42)	1.18 ** (1.10 – 1.27)
Both	1.34 (0.76 – 2.37)	1.55 (1.19 – 2.02)
Race/ethnicities (ref: nHW)		
AA or Black	0.81 ** (0.69 – 0.94)	1.05 (0.98 – 1.13)
Hispanic	1.35 * (1.07 – 1.70)	1.14 (1.00 – 1.30)
Sex (ref: Male)	0.85 *** (0.77 – 0.93)	0.91 * (0.87 – 0.96)
Age	1.07 *** (1.07 – 1.08)	1.02 *** (1.02 – 1.02)
Education	0.98 * (0.96 – 1.00)	0.99 (0.99 – 1.00)
APOE ε+	1.51 *** (1.38 – 1.67)	1.40 *** (1.33 – 1.47)
Seizure* Black or AA	1.30 (0.54 – 3.14)	1.18 (0.78 – 1.80)
TBI* Black or AA	1.51 (0.97 – 2.35)	1.46 (1.02 – 1.80)
Both* Black or AA	1.53 (0.20 – 11.80)	1.77 (0.69 – 4.55)
Seizure *Hispanics	3.52 * (1.06 – 11.71)	2.29 (1.04 – 4.54)

© 2024 Zhu Y et al. *JAMA Network Open.*

TBI*Hispanics	1.61<br>(0.91 – 2.85)	1.72 *<br>(1.28 – 2.30)
Both*Hispanics	1.59<br>(0.20 – 12.44)	0.81<br>(0.30 – 2.22)
Observations	7016	7016
R^2 Nagelkerke	0.080	0.050

* *p<0.05* ** *p<0.01* *** *p<0.001*

© 2024 Zhu Y et al. *JAMA Network Open.*

eTable 4. Subanalysis examining the association between severity and frequency of TBI and cognitive impairment among participants with TBI

	**Progression to CI**
Predictors	*HR(CI)*
TBI frequency (ref: Multiple times)	
Single	1.20 (0.91 – 1.58)
TBI type (ref: Brief unconsciousness)	
Chronic	0.72 (0.23 – 2.26)
Extended unconsciousness	1.08 (0.83 – 1.39)
Without loss of consciousness	0.93 (0.45 – 1.93)
Sex (ref: Male)	0.88 (0.71 – 1.11)
Age	1.06 *** (1.05 – 1.08)
Education	1.01 (0.97 – 1.05)
Race/ethnicities (ref: nHW)	
AA or Black	1.24 (0.82 – 1.88)
Asian	0.90 (0.33 – 2.45)
Hispanic	2.12 ** (1.25 – 3.61)
APOE ε+	1.48 ** (1.16 – 1.88)
Observations	946
R^2 Nagelkerke	0.071

* *p<0.05* ** *p<0.01* *** *p<0.001*

© 2024 Zhu Y et al. *JAMA Network Open.*

eTable 5. Associations between antidepressants generation and cognitive impairment

Predictors	Model without PSW<br>*HR (CI)*	Model with PSW<br>*HR(CI)*
Ref: Neither seizure nor TBI	1.68 *** (1.25 – 2.27)	1.40 ** (1.19 – 1.65)
Seizure	1.29 *** (1.14 – 1.46)	1.25 *** (1.17 – 1.34)
TBI	1.39 (0.82 – 2.36)	1.51 (1.18 – 1.93)
Antidepressant (ref. No antidepressant)		
First-generation	1.20 (0.96 – 1.49)	1.21 * (1.08 – 1.36)
Second-generation	1.36 *** (1.17 – 1.57)	1.30 *** (1.21 – 1.40)
Sex (ref: Male)	0.83 *** (0.76 – 0.91)	0.90 ** (0.85 – 0.94)
Age	1.07 *** (1.07 – 1.08)	1.02 *** (1.02 – 1.02)
Education	0.98 ** (0.96 – 0.99)	0.99 (0.98 – 1.00)
Race/ethnicities (ref: nHW)		
AA or Black	0.87 (0.75 – 1.00)	1.13 * (1.06 – 1.21)
Asian	1.33 (0.96 – 1.82)	1.29 (1.09 – 1.52)
Hispanic	1.48 *** (1.20 – 1.82)	1.26 ** (1.12 – 1.41)
APOE ε+	1.51 *** (1.38 – 1.67)	1.40 *** (1.33 – 1.47)
Observations	7159	7159
R^2 Nagelkerke	0.080	0.052

* *p<0.05* ** *p<0.01* *** *p<0.001*

© 2024 Zhu Y et al. *JAMA Network Open.*

eTable 6. Examining neuropsychiatric symptoms for sensitivity analysis

	Model without PSW	Model with PSW
Predictors	*HR (CI)*	*HR(CI)*
Ref: neither seizure nor TBI		
Seizure	1.72 *** (1.26 – 2.33)	1.44 ** (1.22 – 1.70)
TBI	1.26 *** (1.10 – 1.43)	1.23 *** (1.15 – 1.31)
Both	1.30 (0.72 – 2.36)	1.41 (1.07 – 1.86)
Race/ethnicities (ref: nHW)		
AA or Black	0.83 * (0.71 – 0.96)	1.11 (1.04 – 1.20)
Asian	1.35 (0.97 – 1.89)	1.28 (1.08 – 1.53)
Hispanic	1.39 ** (1.11 – 1.73)	1.20 * (1.07 – 1.36)
Sex (ref: male)	0.86 ** (0.78 – 0.95)	0.94 (0.89 – 0.99)
Age	1.07 *** (1.06 – 1.08)	1.02 *** (1.01 – 1.02)
Education	0.98 ** (0.96 – 0.99)	0.99 (0.99 – 1.00)
APOE ε+	1.49 *** (1.35 – 1.65)	1.38 *** (1.31 – 1.46)
NPIQ symptoms	1.15 *** (1.12 – 1.18)	1.13 *** (1.11 – 1.14)
Observations	6644	6644
R^2 Nagelkerke	0.084	0.080

* *p<0.05* ** *p<0.01* *** *p<0.001*

© 2024 Zhu Y et al. *JAMA Network Open.*

eTable 7. Examining NACC center for sensitivity analysis

	Model without PSW	Model with PSW
Predictors	*HR (CI)*	*HR(CI)*
Ref: neither seizure nor TBI		
Seizure	1.65 ** (1.22 – 2.23)	1.40 ** (1.19 – 1.65)
TBI	1.26 *** (1.11 – 1.43)	1.22 *** (1.14 – 1.30)
Both	1.37 (0.81 – 2.33)	1.50 * (1.17 – 1.92)
Sex (ref: Male)	0.82 *** (0.74 – 0.90)	0.87 ** (0.83 – 0.92)
Age	1.08 *** (1.07 – 1.09)	1.03 *** (1.02 – 1.03)
Education	0.97 *** (0.95 – 0.98)	0.98 ** (0.97 – 0.99)
Race/ethnicities (ref: nHW)		
AA or Black	0.95 (0.80 – 1.12)	1.17 * (1.08 – 1.27)
Asian	1.30 (0.94 – 1.80)	1.22 (1.03 – 1.44)
Hispanic	1.40 ** (1.12 – 1.76)	1.17 (1.04 – 1.33)
APOE ε+	1.51 *** (1.38 – 1.67)	1.42 *** (1.35 – 1.49)
NACCADC [354]	0.59 * (0.39 – 0.91)	0.52 *** (0.41 – 0.64)
NACCADC [490]	1.76 ** (1.17 – 2.66)	1.53 * (1.25 – 1.88)
NACCADC [911]	0.95 (0.29 – 3.07)	0.90 (0.52 – 1.56)
NACCADC [943]	3.95 *** (1.91 – 8.15)	2.39 * (1.72 – 3.32)

© 2024 Zhu Y et al. *JAMA Network Open.*

NACCADC [1018]	4.01 *** (2.21 – 7.30)	3.74 *** (2.75 – 5.08)
NACCADC [1354]	0.59 ** (0.39 – 0.88)	0.61 ** (0.49 – 0.75)
NACCADC [1416]	1.04 (0.54 – 1.98)	0.98 (0.68 – 1.39)
NACCADC [2096]	0.41 *** (0.27 – 0.64)	0.42 *** (0.34 – 0.52)
NACCADC [2125]	1.68 (0.60 – 4.71)	1.83 (1.04 – 3.21)
NACCADC [2289]	0.75 (0.47 – 1.18)	0.79 (0.62 – 0.99)
NACCADC [2578]	0.80 (0.56 – 1.16)	0.76 (0.63 – 0.92)
NACCADC [2958]	0.96 (0.64 – 1.46)	0.82 (0.66 – 1.02)
NACCADC [3630]	0.42 ** (0.25 – 0.71)	0.48 ** (0.36 – 0.62)
NACCADC [3697]	2.88 *** (1.59 – 5.21)	1.95 ** (1.46 – 2.61)
NACCADC [4032]	1.01 (0.55 – 1.85)	1.09 (0.79 – 1.50)
NACCADC [4347]	0.49 *** (0.32 – 0.75)	0.48 *** (0.38 – 0.60)
NACCADC [4935]	0.94 (0.63 – 1.40)	0.92 (0.75 – 1.14)
NACCADC [4967]	0.89 (0.58 – 1.37)	0.83 (0.67 – 1.03)
NACCADC [5310]	0.43 ** (0.25 – 0.73)	0.55 * (0.42 – 0.72)
NACCADC [5452]	1.63 * (1.12 – 2.37)	1.45 * (1.20 – 1.75)
NACCADC [5783]	0.75 (0.53 – 1.06)	0.65 ** (0.54 – 0.77)

© 2024 Zhu Y et al. *JAMA Network Open.*

NACCADC [5897]	1.23<br>(0.61 – 2.47)	1.50<br>(1.08 – 2.10)
NACCADC [6061]	1.31<br>(0.91 – 1.88)	1.13<br>(0.94 – 1.35)
NACCADC [6499]	1.12<br>(0.70 – 1.79)	1.12<br>(0.89 – 1.41)
NACCADC [6518]	1.52 *<br>(1.04 – 2.20)	1.25<br>(1.04 – 1.51)
NACCADC [6713]	1.19<br>(0.75 – 1.88)	1.10<br>(0.88 – 1.37)
NACCADC [8354]	1.58 *<br>(1.02 – 2.45)	1.22<br>(0.98 – 1.51)
NACCADC [8361]	0.78<br>(0.52 – 1.16)	0.70 *<br>(0.57 – 0.87)
NACCADC [8646]	0.80<br>(0.57 – 1.13)	0.69 *<br>(0.58 – 0.82)
NACCADC [8658]	1.07<br>(0.76 – 1.50)	0.89<br>(0.75 – 1.05)
NACCADC [8660]	1.90<br>(0.26 – 13.83)	1.66<br>(0.50 – 5.46)
NACCADC [8683]	1.62 **<br>(1.13 – 2.34)	1.11<br>(0.92 – 1.34)
NACCADC [8974]	0.62 *<br>(0.39 – 0.99)	0.57 **<br>(0.45 – 0.72)
NACCADC [9637]	0.74<br>(0.44 – 1.24)	0.86<br>(0.66 – 1.10)
NACCADC [9661]	1.07<br>(0.69 – 1.65)	0.83<br>(0.66 – 1.04)
Observations	7159	7159
R^2 Nagelkerke	0.117	0.149

* *p<0.05* ** *p<0.01* *** *p<0.001*

© 2024 Zhu Y et al. *JAMA Network Open.*

eTable 8. Associations between seizure and TBI and progression to cognitive impairment

	**Fine and Grey Model**	**Model using optimal full matching**
Predictors	*HR (CI)*	*HR(CI)*
Ref: neither seizure nor TBI		
Seizure	1.65 *** (1.22 – 2.23)	1.97 *** (1.51 – 2.57)
TBI	1.32 *** (1.17 – 1.49)	1.23 * (1.09 – 1.39)
Both	1.41 (0.83 – 2.39)	2.24 * (1.56 – 3.23)
Race/ethnicities (ref: nHW)		
Black or AA	0.84 * (0.73 – 0.97)	0.94 (0.82 – 1.07)
Asian	1.35 (0.98 – 1.86)	0.90 (0.63 – 1.28)
Hispanic	1.47 *** (1.19 – 1.81)	1.19 (0.96 – 1.48)
Sex (ref: Male)	0.89 ** (0.81 – 0.97)	1.00 (0.91 – 1.10)
Age	1.05 *** (1.05 – 1.06)	1.02 ** (1.01 – 1.02)
Education	0.98 * (0.97 – 1.00)	1.00 (0.98 – 1.01)
APOE ε+	1.49 *** (1.35 – 1.64)	1.54 *** (1.40 – 1.69)
Observations	7159	7159
R^2 Nagelkerke	0.078	0.045

* *p<0.05* ** *p<0.01* *** *p<0.001*

© 2024 Zhu Y et al. *JAMA Network Open.*

eTable 9. The number of users of medications by racial and ethnic groups

	AA or Black (N=1036)	Asian (N=143)	Hispanic (N=332)	White (N=5648)
Antidepressant				
No	962 (92.9%)	138 (96.5%)	280 (84.3%)	4615 (81.7%)
Yes	68 (6.6%)	4 (2.8%)	52 (15.7%)	963 (17.1%)
Missing	6 (0.6%)	1 (0.7%)	0 (0%)	70 (1.2%)
Frist generation users	19(0.2%)	0(0%)	11(3.3%)	152 (2.7%)
Second generation users	43(4.2%)	4(2.7%)	37(11.14%)	709(12.6%)
Antiseizure medication				
No	992 (95.8%)	139 (97.2%)	312 (94.0%)	5415 (95.9%)
Yes	44 (4.2%)	4 (2.8%)	20 (6.0%)	233 (4.1%)
Antipsychotic agent				
No	1028 (99.2%)	142 (99.3%)	330 (99.4%)	5557 (98.4%)
Yes	2 (0.2%)	0 (0%)	2 (0.6%)	21 (0.4%)
Missing	6 (0.6%)	1 (0.7%)	0 (0%)	70 (1.2%)
Anxiolytic, sedative, or hypnotic agent.				
No	966 (93.2%)	131 (91.6%)	301 (90.7%)	4904 (86.8%)
Yes	64 (6.2%)	11 (7.7%)	31 (9.3%)	674 (11.9%)
Missing	6 (0.6%)	1 (0.7%)	0 (0%)	70 (1.2%)

© 2024 Zhu Y et al. *JAMA Network Open.*
