# Supplementary material for: Traumatic Brain Injury, Seizures, and Cognitive Impairment Among Older Adults
Source: JAMA Netw Open. 2024 Aug 8;7(8):e2426590. doi: 10.1001/jamanetworkopen.2024.26590 (PMC11310819; doi:10.1001/jamanetworkopen.2024.26590)
Supplement: Supplement 2. — Data Sharing Statement [file jamanetwopen-e2426590-s002.pdf]

Data Sharing Statement

Zhu. Traumatic Brain Injury, Seizures, and Cognitive Impairment Among Older Adults. *JAMA Netw Open*. Published August 08, 2024. doi:10.1001/jamanetworkopen.2024.26590

Data
Data available: No

Additional Information
Explanation for why data not available: Data can be made available upon reasonable request.
